# Differential impact of BRAFV600E isoforms on tumorigenesis in a zebrafish model of melanoma

**DOI:** 10.1186/s13578-023-01064-w

**Published:** 2023-07-01

**Authors:** Raffaella De Paolo, Samanta Sarti, Sara Bernardi, Francesco Cucco, Andrea Tavosanis, Letizia Pitto, Laura Poliseno

**Affiliations:** 1 Institute of Clinical Physiology, CNR, Pisa, Italy; 2 Oncogenomics Unit, Core Research Laboratory (CRL), ISPRO, Via Moruzzi 1, 56124 Pisa, Italy; 3 Present Address: Department of Radiation Oncology, Columbia University Irving Medical Center, New York, USA; 4 Present Address: Department of Molecular Medicine and Neurobiology, IRCCS Fondazione Stella Maris, Pisa, Italy; 5 Present Address: San Raffaele Telethon Institute for Gene Therapy, IRCCS San Raffaele Scientific Institute, Milan, Italy

**Keywords:** Zebrafish, Melanoma modeling, BRAFV600E-ref, BRAFV600E-X1, 3’UTR

## Abstract

BRAFV600E comes as two main splicing variants. The well-studied ref isoform and the recently discovered X1 isoform are co-expressed in cancer cells and differ in terms of 3’UTR length and sequence, as well as C-term protein sequence. Here, we use a melanoma model in zebrafish to study the role played by each isoform in larval pigmentation, nevi formation, and their progression into melanoma tumours. We show that both BRAFV600E-ref and BRAFV600E-X1 proteins promote larval pigmentation and nevi formation, while melanoma-free survival curves performed in adult fish indicate that BRAFV600E-ref protein is a much stronger melanoma driver that BRAFV600E-X1 protein. Crucially, we also show that the presence of the 3’UTR suppresses the effect of ref protein. Our data highlight the necessity to undertake a systematic study of BRAFV600E isoforms, in order to uncover the full spectrum of their kinase-(in)dependent and coding-(in)dependent functions, hence to develop more informed strategies for therapeutic targeting.

**Supplementary Information:**

The online version contains supplementary material available at 10.1186/s13578-023-01064-w.

**Dear Editor,**

Melanoma originates from melanocytes and is responsible for the highest mortality among skin cancers. As a result, significant research has been dedicated to its study, and zebrafish models recapitulating the most common genetic alterations have offered several notable contributions to this field [1].

A specific characteristic of melanoma is the recurrent overactivation of the ERK pathway, most often because of the BRAFV600E mutation, which is now routinely targeted by specific inhibitors approved for use by the FDA [2]. The *BRAF* gene is characterised by several splicing variants, and while some of them associated with drug resistance have been well investigated, comparatively little is known about their physiological regulation. Different protein isoforms may exhibit different biological properties, including catalytic capacity, subcellular localization, and protein–protein interaction. Similarly, distinct mRNA isoforms may gain unique binding sites for miRNAs and RNA-binding proteins. In short, investigating the landscape of BRAF isoforms may reveal kinase- and coding-(in)dependent functions that directly or indirectly affect melanoma onset, progression or escape from antineoplastic treatments.

We recently reported that irrespectively of its mutational status human *BRAF* is expressed as a mix of *ref*, *X1* and *X2* splicing variants. The *reference* (*ref*) isoform is composed of 18 exons. Exon 18 contains the STOP codon and a short 3’UTR (~ 100nt). The *X1* isoform is composed of a shorter version of exon 18, which is spliced with a downstream exon 19. This last exon contains the STOP codon and a very long 3’UTR (~ 7000nt). The X2 isoform lacks exon 18, with exon 17 directly spliced with exon 19. Also in this case, exon 19 contains the STOP codon, through a different frame, and the very long 3’UTR [3] (see also Additional file 1 : Fig. S1). These isoforms are always co-expressed in cancer cells, with *X1* much more expressed than *ref* and *X2* [3]. Interestingly, *ref* and *X1* 3’UTRs are subjected to post-transcriptional regulation by distinct groups of microRNAs and RBPs. They positively or negatively affect mRNA stability or translation, and, consequently, contribute to fine tune the output of MAPK signaling pathway [4–6]. In terms of proteins, ref and X1 differ at the C-terminal domain (ref: –GYG**AFPVH** vs. X1: –GYG**EFAAFK**), are both endowed with kinase activity, and together account for the known oncogenic features displayed by BRAFV600E in melanoma cells [3, 7] (see also Additional file 1 : Fig. S1). Conversely, X2 protein is quite unstable and rapidly undergoes proteasome-dependent degradation, due to the presence of K_739_ residue in its C-terminal domain [3].

Here, we use a p53-mutated tumour-prone zebrafish line to compare the BRAFV600E-ref isoform with the BRAFV600E-X1 isoform. We found that BRAFV600E-ref protein is a much stronger melanoma driver than BRAFV600E-X1 protein, but this difference is abolished in presence of the 3’UTR.

Currently, five annotated protein sequences are documented for BRAF and two of them are included in the consensus coding sequence database (CCDS): #220 and #204. Comparing the most updated annotation with our own previous studies [3], we conclude that the ref isoform corresponds to #220 and the X1 isoform corresponds to #204, while X2 is not currently annotated in the CCDS. Current mRNA sequences are: *NM_004333.6* and* ENST00000646891.2* for *BRAF-ref*, *NM_001354609.2* and *ENST00000496384.7* for *BRAF-X1*, and *NM_001378468.1* for *BRAF-X2*.

To the best of our knowledge, all in vivo cancer models available so far make use of BRAFV600E-ref cds [1]. In particular, the expression of Myc-tagged BRAFV600E oncogene in the melanocytic lineage of zebrafish leads to the formation of nevi that progress to melanoma in case of p53 deficiency (*Tg(mitfa:BRAFV600E-Myc);p53(lf)* line [8]). Building up on this, we have developed a model system that allows to compare BRAFV600E-ref versus X1 cds isoforms, as well as to investigate the contribution of the respective 3’UTRs. Specifically, we generated plasmids expressing ref or X1 cds, with or without their 3’UTR (Fig. 1a, see also Additional files 2, 3, 4). As reported in [8], we used *mitfa* promoter to confine the expression of the oncogene in melanocytes. However, we avoided fusing the proteins’ C-terminal domain to a tag, as that would compromise our ability to discriminate their different functionalities. As far as 3’UTRs are concerned, their size was chosen based on our previous analysis: 121nt for *ref* and 7163nt for *X1* [3]. Also, we relied on the expression of a cardiac eGFP reporter to screen for plasmid integration. Finally, plasmid cloning was performed using Tol2kit, so that the DNA portion of the plasmid located between Tol2 elements gets effectively integrated in the zebrafish genome through Tol2-mediated transgenesis.

**Fig. 1:**
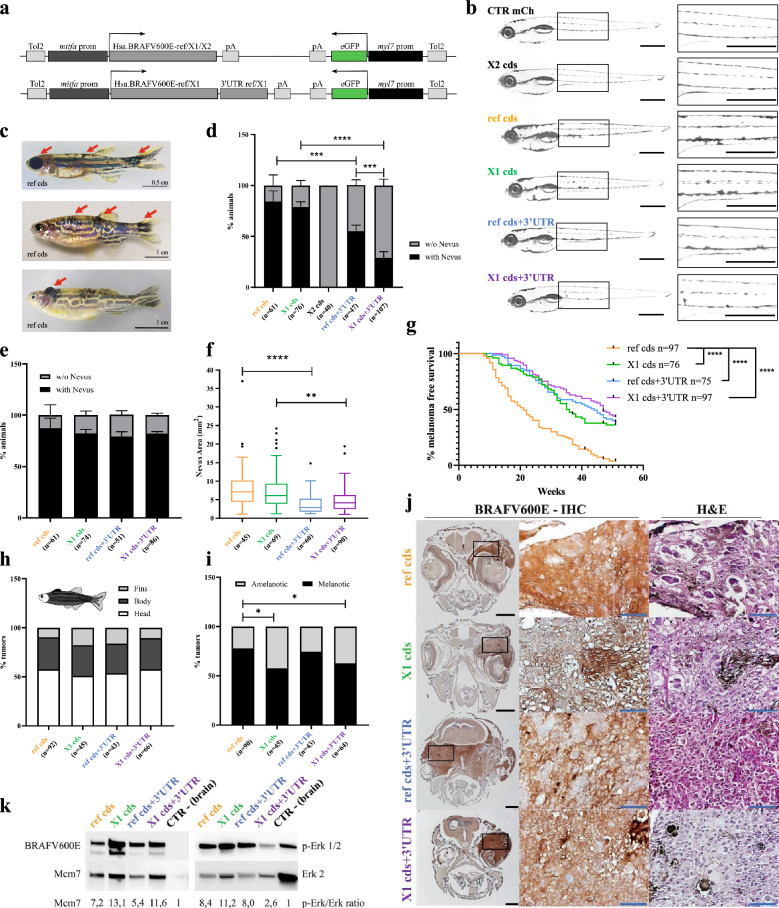
Impact of BRAFV600E isoforms on melanomagesis in zebrafish. **a** Schematic representation of the plasmids that express human BRAFV600E isoforms (*upper*, coding sequence (ref cds, X1 cds, and X2 cds); *lower*, ref cds + 3’UTR, and X1 cds + 3’UTR) under the control of *mitfa* promoter (*mitfa* prom), and eGFP reporter (green) under the control of cardiac *myl7* promoter (*myl7* prom). Tol2: minimal elements of Tol2 transposon; pA: polyA tail. **b** Pigmentation pattern in larvae at 5dpf. Larvae that were injected at 1-cell stage with ref and X1 cds plasmids show increased number or abnormal appearance of pigmented spots. Left: lateral view; right: lateral zoom view. A 5dpf *Tg(mitfa:mCherry,myl7:eGFP);p53(lf)* larva is shown as negative control (CTR mCh). Scale bars: 500 μm. **c** Representative examples of a juvenile fish with nevi (*upper*, red arrows), an adult fish with nevi (*middle*, red arrows), and an adult fish with a melanoma tumor (*lower*, red arrow). **d** Percentage of juvenile fish with a nevus. Nevi develop in higher percentage in juveniles injected with ref and X1 cds plasmids. Data are expressed as mean ± SEM. The number of juvenile fish per experimental condition (n) is reported in brackets. Differences were analyzed using Fisher’s exact test. **e** Percentage of adult fish with a nevus. Data are expressed as mean ± SEM. The number of adult fish per experimental condition (n) is reported in brackets. Differences were analyzed using Fisher’s exact test. No difference reaches statistical significance. **f** Size of nevi in adult fish (3 months of age). Adults injected with ref and X1 cds plasmids show nevi characterized by bigger area. Data are expressed as mean ± SEM. The number of adult fish per experimental condition (n) is reported in brackets. Differences were analyzed using Kruskal–Wallis (Dunn’s) test. **g** One-year long melanoma-free survival curves uncover ref cds as the most potent melanoma driver compared to X1 cds, ref cds + 3’UTR, and X1 cds + 3’UTR. The number of adult fish per experimental condition (n) is reported in brackets. Differences were analyzed using log-rank (Mantel-Cox) test. **h, i** Macro features of melanoma tumors developed in adults. **h** Tumors localization. **i** Presence of pigmentation. Melanotic tumors develop at higher percentage in fish injected with ref cds and ref cds + 3’UTR plasmids. The number of adult fish per experimental condition (n) is reported in brackets. Differences were analyzed using Fisher’s exact test. **j** Representative images of BRAFV600E immunohistochemistry staining (*left*) and Hematoxylin and Eosin staining (H&E, *right*) performed on melanoma tumors in adult fish. Black scale bar: 500 μm; blue scale bar: 90 μm. **k** Western blot detection of BRAFV600E (left, *upper*), Mcm7 (left, *lower*) p-Erk 1/2 (right, *upper*) and Erk 2 (right, *lower*) in representative melanoma tumors excised from adult fish. Brain tissue is used as negative control (CTR–). The quantification of Mcm7 and p-Erk/Erk ratio is reported at the bottom of the panels and is expressed as fold change over the negative control. Color coding: yellow: ref cds; green: X1 cds; black: X2 cds; blue: ref cds + 3’UTR; purple: X1 cds + 3’UTR. Statistically significant differences are indicated with asterisks: *P < 0.05, **P < 0.01, ***P < 0.001, ****P < 0.0001

Plasmids were co-injected with *Transposase* mRNA in 1-cell embryos of the p53-mutant and tumor-prone ZDB-ALT-050428-2 (*p53(lf)*) zebrafish line. At 24 h post fertilization (hpf) we selected successfully injected embryos based on the presence of a green heart. We also validated the expression of all *BRAFV600E* isoforms, including coding sequence (cds)-only and coding sequence plus 3’UTR (cds + 3’UTR) transcripts (Additional file 1 : Fig. S2a, b). mRNA levels were quantified at both 24hpf and 5 days post fertilization (dpf) (Additional file 1 : Fig. S2c, d). Interestingly, we noticed that *X1* cds + 3’UTR expression is much higher compared to *ref* cds + 3’UTR, in agreement with the data we reported on melanoma samples and cell lines [3].

We thus proceeded to the analysis of the biological consequences of BRAFV600E isoform overexpression. The mosaic condition exhibits altered pigmentation starting at the larval stage (5dpf). This phenotype is most apparent for recipients of the ref and X1 cds plasmids, while cds + 3’UTR recipients display a milder phenotype (Fig. 1b). Such trend is maintained at the juvenile stage, in terms of percentage of animals showing development of a nevus (Fig. 1c, upper, d and Additional file 1 : Fig. S3a), and upon reaching adulthood, in terms of nevi size (Fig. 1c, middle, e, f and Additional file 1 : Fig. S3b). As expected, the X2 variant shows no impact at any stage of development. Reflecting the fact that nevi number and size are important clinical prognostic factors in human, we recorded melanoma-free survival curves at the adult stage over a 1-year observation period, focusing on the comparison between ref and X1. Strikingly, we found that ref cds is a much stronger melanoma driver than all the others (Fig. 1c, lower, g and Additional file 1 : Fig. S4), without affecting the development of tumors across the fish body (Fig. 1h), but potentially enhancing the emergence of melanotic tumors (Fig. 1i).

It remains to be elucidated how the few amino acids distinguishing BRAFV600E-ref and –X1 have no impact on nevi development (compare yellow and green in Fig. 1b, d–f), while they have such a dramatic impact on nevi transformation into melanoma (compare yellow and green lines in Fig. 1g). Major alterations in BRAFV600E protein levels or ability to activate ERK pathway can be excluded (Fig. 1j, k and Additional file 1 : Fig. S5). However, more detailed analyses in ad hoc experimental settings may reveal subtle differences in substrate preferences. Another possibility is that the different C-terminal domains exert kinase-independent functions, such as interactions with different sets of proteins and activation of different signaling pathways, or are engaged in different regulatory mechanisms.

The milder effect exhibited by the cds + 3’UTR plasmids is likely because 3’UTRs are intrinsically devoted to regulation and tuning of gene expression [9]. Nevertheless, several puzzling issues remain: why the short *ref* 3’UTR mildly affects nevi development (compare yellow and blue in Fig. 1b, d–f), but has a dramatic impact on nevi transformation into melanoma, completely reversing the effect of ref cds (compare yellow and blue lines in Fig. 1g)? Conversely, why the long *X1* 3’UTR severely delays nevi development (compare green and purple in Fig. 1b, d–f), in spite of the fact that it ensures higher expression levels to *X1* cds + 3’UTR mRNA (compare green and purple bars in Additional file 1 : Fig. S2c, d), and then it has a negligible impact on nevi transformation into melanoma (compare green and purple lines in Fig. 1g)? In general terms, we can speculate that the impact of the *X1* 3’UTR is mild, being the X1 cds a weak melanoma driver per se, while the *ref* 3’UTR contributes to tame the strong oncogenicity of the ref cds. However, the mechanistic details underlying each biological outcome, in each phase of fish life, remain to be uncovered taking advantage of the more homogeneous genetic background provided by stable transgenic lines.

In summary, in this work we show that different cds and 3’UTR sequences of BRAFV600E differentially affect tumorigenesis in a zebrafish melanoma model. This experimental data urge to undertake a systematic analysis of BRAFV600E isoforms beyond the ref kinase, which so far has catalyzed the attention of the melanoma scientific community. Populating the field of kinase- and coding-(in)dependent functions of BRAFV600E isoforms can in turn prove instrumental to achieve a more informed, hence more effective, therapeutic targeting. Since ref and X1 isoforms are co-expressed across cancer types, our data also suggests generating and testing appropriate constructs in other experimental models of (BRAFV600E-driven) cancer types. Finally, it highlights the necessity to include untranslated regions, as they can heavily modify the biological outcome.

Transcending the boundaries of cancer biology, our findings indicate that *BRAF* gene has evolved significantly: the older X1 protein is present in the ancient vertebrate lamprey, while the younger ref protein appears in marsupials (wallaby) (Additional file 1 : Fig. S6). Alternative splicing is a key component of biological complexity, and it is gaining momentum for its role in adaptation and evolution [10]. Therefore, we need to understand how and when ref isoform originated. We also need to discover the specific functions it carries out and whether its low levels represent a failsafe mechanism, since it is so oncogenic when mutated.

## Supplementary Information

**Additional file 1.** Supplementary figures.

**Additional file 2.** Supplementary methods.

**Additional file 3.** Supplementary material. Sequence of ref cds, X1 cds, X2 cds, ref 3’UTR, X1 3’UTR.

**Additional file 4.** Supplementary Table 1. Primer sequence and use.

## Data Availability

Not applicable.

## Abbreviations

3’UTR: 3’ Untranslated region
cds: Coding sequence
dpf: Days post fertilization
FDA: Food and drug administration
hpf: Hours post fertilization
miRNA: MicroRNA
pA: Poly A
RBP: RNA binding protein
ref: Reference
