# Supplementary material for: Differential impact of BRAFV600E isoforms on tumorigenesis in a zebrafish model of melanoma
Source: Cell Biosci. 2023 Jul 1;13:121. doi: 10.1186/s13578-023-01064-w (PMC10314448; doi:10.1186/s13578-023-01064-w)
Supplement: Supplementary file 1 — Additional file 1. Supplementary figures. [file 13578_2023_1064_MOESM1_ESM.pdf]

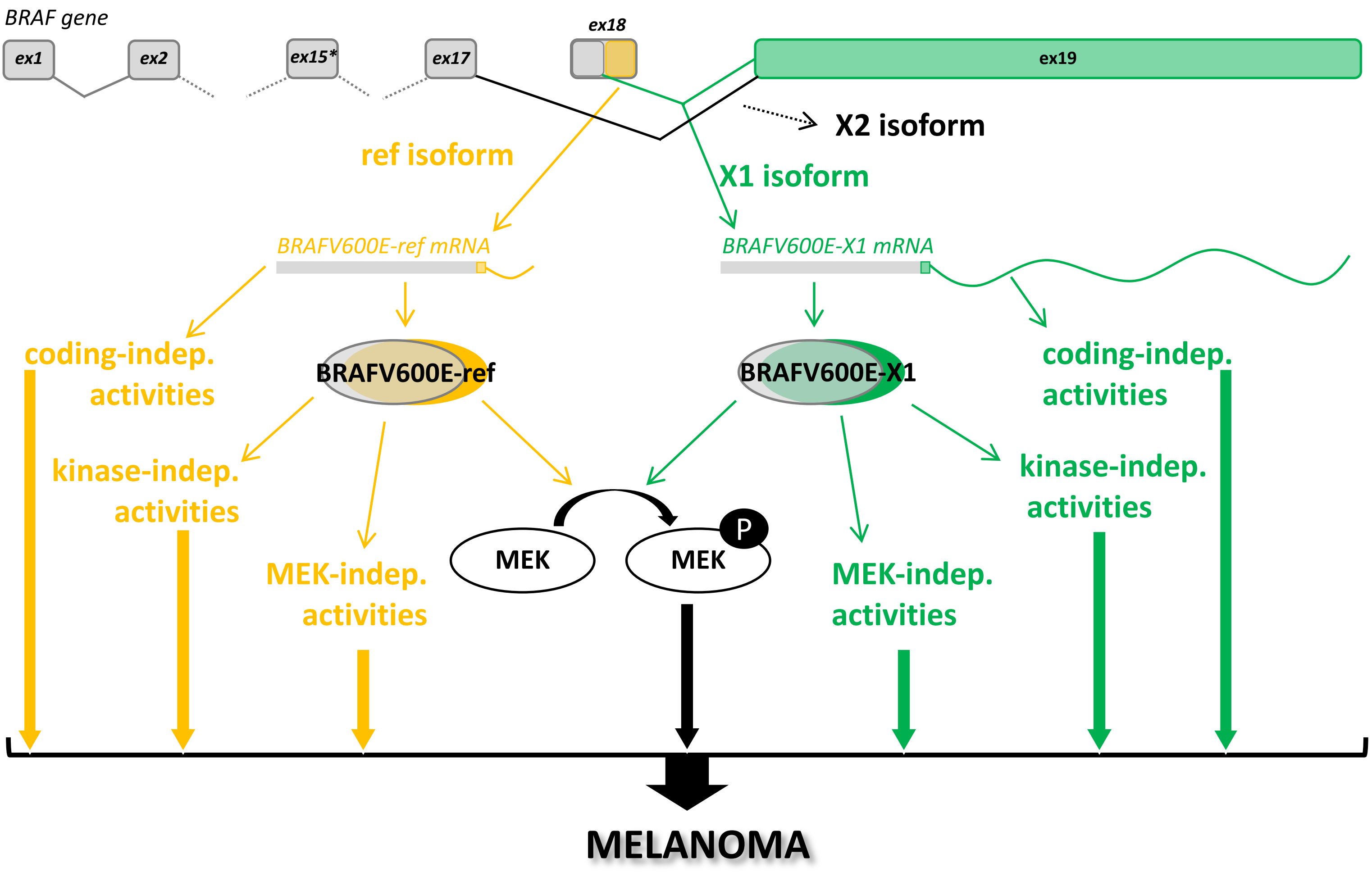

Supplementary Figure 1. Cartoon summarizing *BRAF* gene expression and functions.
The cartoon represents *BRAF* exons, as well as ref (yellow) and X1 (green) isoforms (mRNAs and proteins). In addition, the cartoon summarizes the functions possibly exerted by BRAF mRNA and protein isoforms, besides the well-known ability of BRAF kinase to phosphorylate MEK.
Exon 15, which encodes for the V600E mutation, is highlighted with an asterisk.

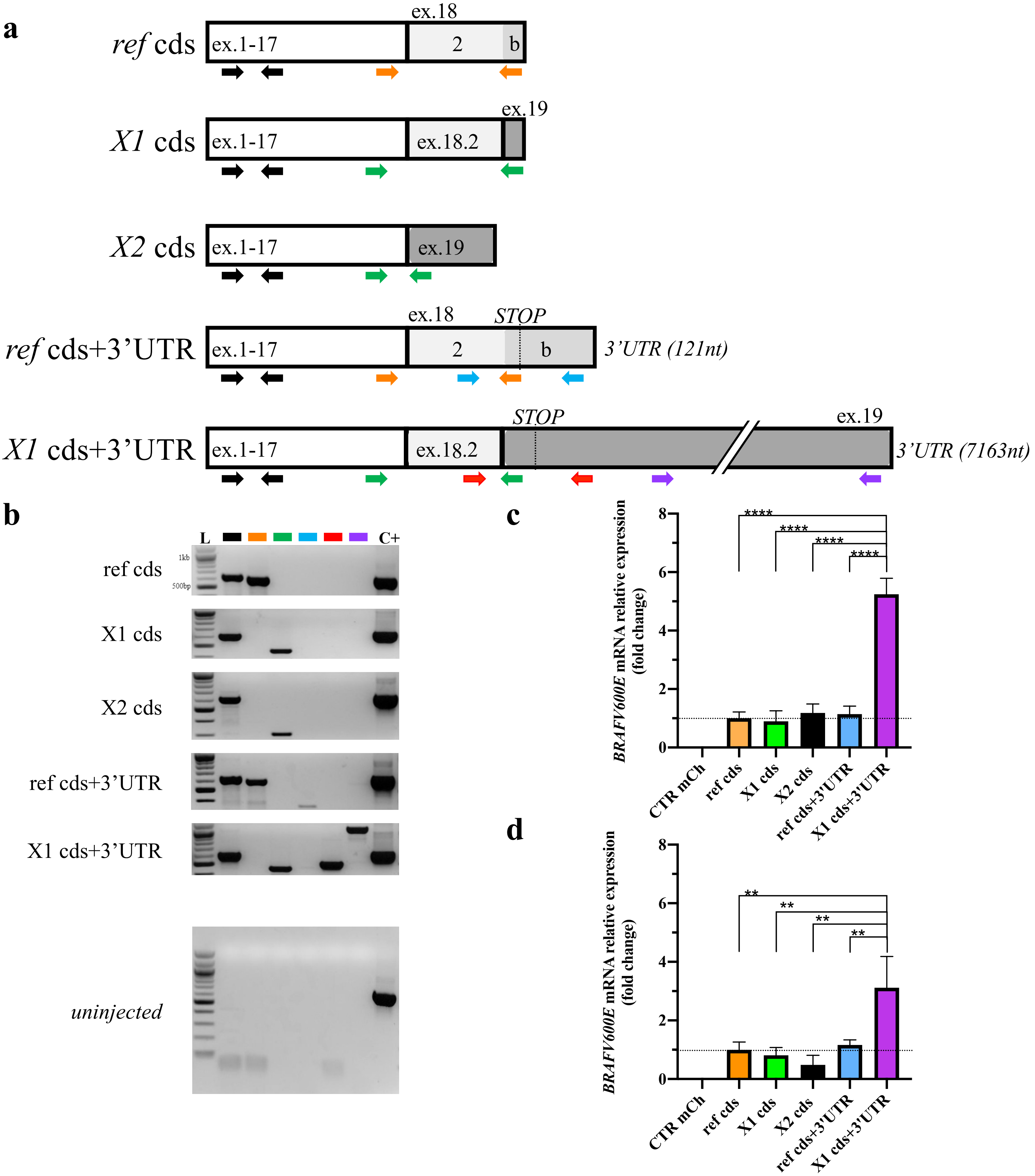

Supplementary Figure 2. Expression levels of *BRAFV600E* isoforms in *p53(lf)* embryos and larvae.
(a-b) PCR analysis. **(a)** Cartoon summarizing the position of PCR primers (colored arrows) used to determine the correct expression of cds and 3'UTR sequences of *BRAFV600E* mRNA isoforms. Exons (ex) are not in scale. **(b, upper)** Representative results of the PCR performed at 24hpf on *p53(lf)* embryos. These embryos were injected at 1-cell stage with the indicated plasmids. Primer pairs used are color-coded as in **a**. L: 100bp DNA ladder. C+: *actb1* exon-spanning primers are used as positive control. These primers amplify a 600bp band on cDNA and a 900bp band on genomic DNA. **(b, lower)** No PCR amplification, except for the positive control, are observed in uninjected *p53(lf)* embryos, ensuring the specificity of primer pairs for exogenous human *BRAF* over endogenous zebrafish *Braf*.
(c-d) qRT-PCR analysis. qRT-PCR was performed at 24hpf **(c)** and at 5dpf **(d)** on *p53(lf)* embryos/larvae injected at 1-cell stage with the indicated plasmids. *Tg(mitfa:mCherry,myl7:eGFP);p53(lf)* embryos/larvae are used as negative control. Data are expressed as mean ± SEM. Differences were analyzed using one-way ANOVA (Tukey's) test. Statistically significant differences are indicated with asterisks: **P<0.01, ****P<0.0001. qRT-PCR: quantitative Real Time Polymerase Chain Reaction; hpf: hours post fertilization; dpf: days post fertilization.

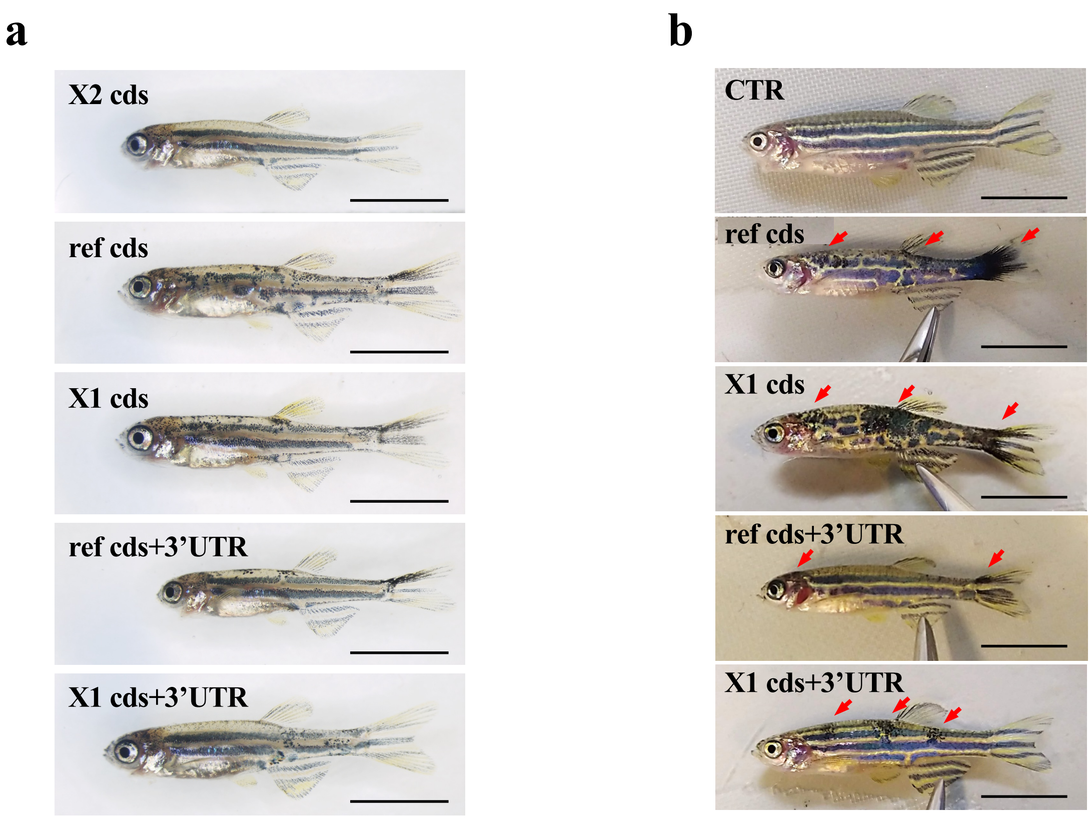

Supplementary Figure 3. Representative nevi in juvenile and adult *p53(lf)* fish injected with the indicated plasmids.
(a) Representative nevi in juvenile fish. A fish injected with X2 cds construct is used as negative control. Scale bar: 0.5cm.
(b) Representative nevi (red arrows) in adult fish (3 months of age). An uninjected fish is used as negative control (CTR). Scale bar: 1cm.

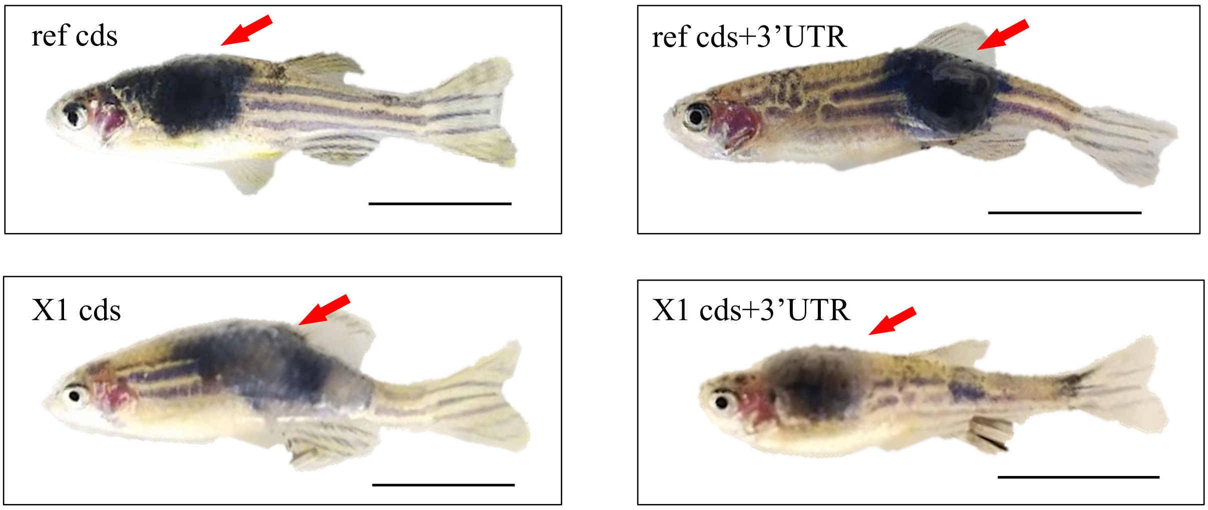

Supplementary Figure 4. Representative melanoma tumors in adult *p53(lf)* fish injected with the indicated plasmids. Tumors are indicated with red arrows. Scale bar: 1cm.

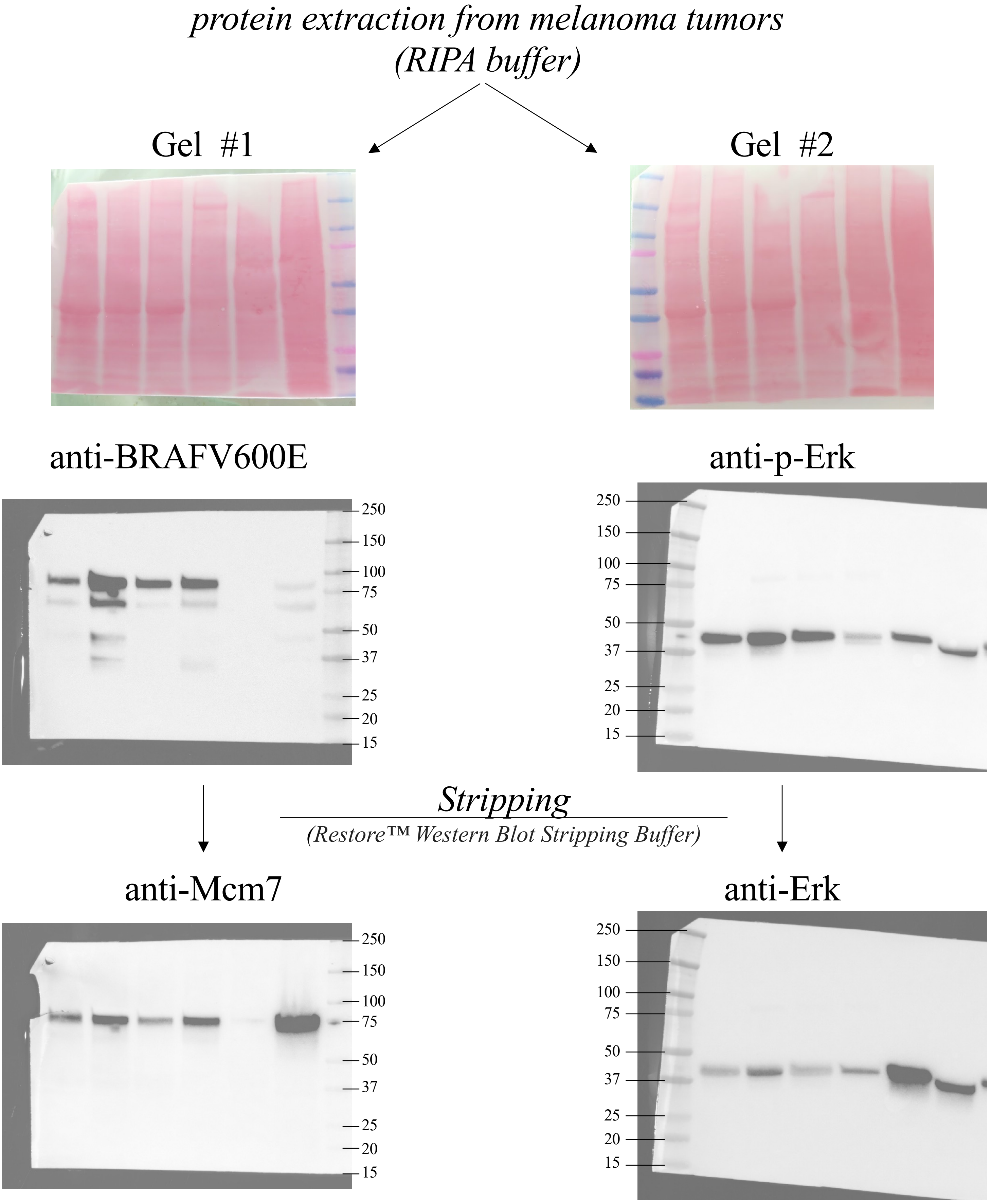

Supplementary Figure 5. Protein loading and whole blots of the western blot presented in Fig.1k.

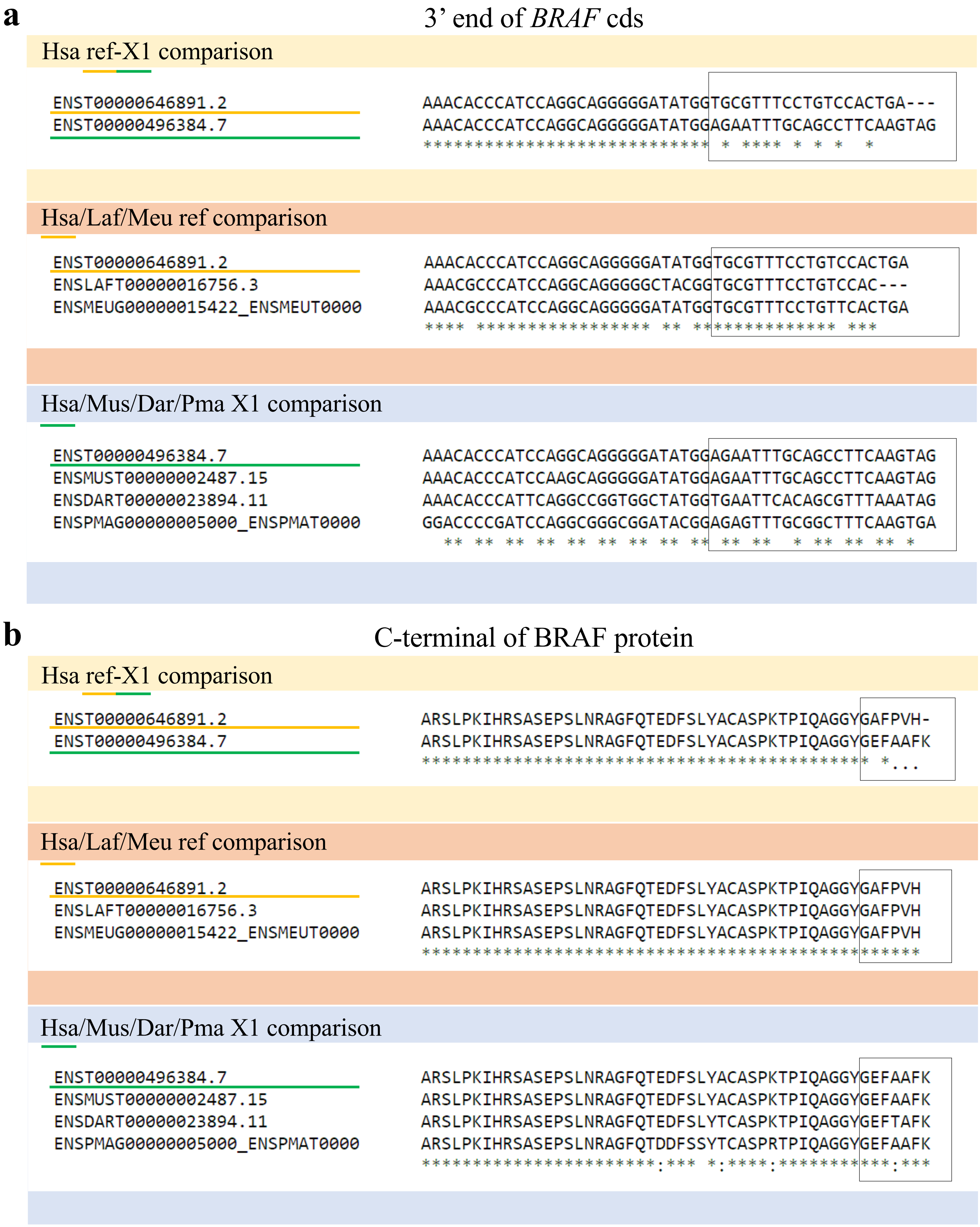

Supplementary Figure 6. Alignment of the 3' end of *BRAF* cds and the C-terminal of BRAF protein.
(a) Alignment of the 3' end of *BRAF* cds. *Upper:* alignment of *ref* (yellow underline) and *X1* (green underline) sequence of human *BRAF*. *Middle:* alignment of human *BRAF-ref* (yellow underline; *Homo sapiens* Hsa) with elephant *Braf* (*Loxodonta africana* Laf) and wallaby *Braf* (*Macropus eugenii* Meu). *Lower:* alignment of human *BRAF-X1* (green underline; *Homo sapiens* Hsa) with mouse *Braf* (*Mus musculus* Mus), zebrafish *braf* (*Danio rerio* Dar), and lamprey *braf* (*Petromyzon marinus* Pma). The boxes on the right highlight that DNA/RNA sequences are not conserved.
(b) Alignment of the C-terminal of BRAF protein. *Upper:* alignment of ref (yellow underline) and X1 (green underline) sequence of human BRAF. *Middle:* alignment of human BRAF-ref (yellow underline; *Homo sapiens* Hsa) with elephant BRAF (*Loxodonta africana* Laf) and wallaby BRAF (*Macropus eugenii* Meu). *Lower:* alignment of human BRAF-X1 (green underline) with mouse Braf (*Mus musculus* Mus), zebrafish Braf (*Danio rerio* Dar), and lamprey Braf (*Petromyzon marinus* Pma). The boxes on the right highlight the differences in protein sequence between human ref and X1 isoforms, the conservation of ref protein isoform in mammals across wallaby, elephant and human, as well as the conservation of X1 protein isoform outside mammals and across lamprey, zebrafish, mouse, and human.

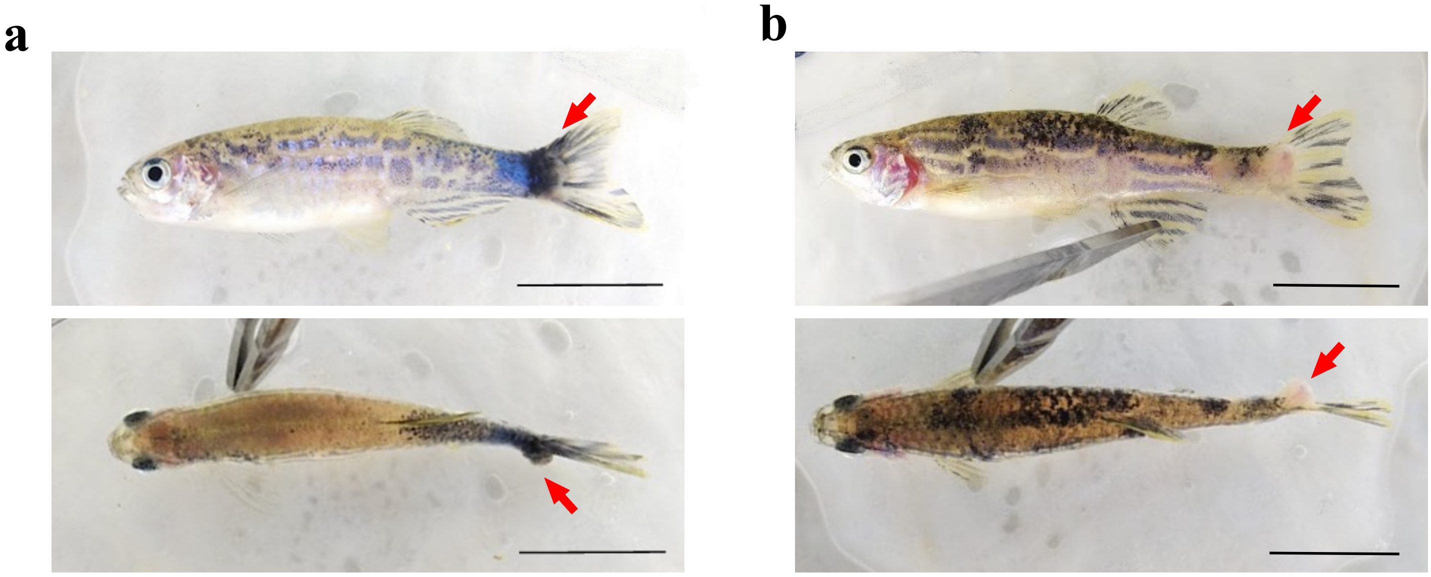

Supplementary Figure 7. Phenotypical analysis.
(a) Representative example of a melanotic tumor (red arrow) in lateral (*upper*) and dorsal (*lower*) view. Scale bar: 1cm.
(b) Representative example of an amelanotic tumor (red arrow) in lateral (*upper*) and dorsal (*lower*) view. Scale bar: 1cm.
