# Supplementary material for: Differential impact of BRAFV600E isoforms on tumorigenesis in a zebrafish model of melanoma
Source: Cell Biosci. 2023 Jul 1;13:121. doi: 10.1186/s13578-023-01064-w (PMC10314448; doi:10.1186/s13578-023-01064-w)
Supplement: Supplementary file 2 — Additional file 2. Supplementary methods. [file 13578_2023_1064_MOESM2_ESM.docx]

**SUPPLEMENTARY METHODS**

**Zebrafish husbandry**

The zebrafish facility at CNR-IFC has been authorized by the Italian Ministry of Health (authorization #297/2012-A, issued on December 21, 2012) and by the Municipality of Pisa (authorization #DN-16/504, issued on June 7, 2013). Zebrafish (*Danio rerio*) experiments were carried out in accordance with the European Union guidelines for animal welfare [European Communities Council Directive of September 22, 2010 (2010/63/ UE)]. All experimental protocols were approved by the Italian Ministry of Health (authorization #383/2020-PR). Zebrafish were raised and maintained on a 14h/10h light/dark cycle at 28.5°C, in a zebrafish housing system (Tecniplast) under pH- and salinity-controlled conditions. Embryos were obtained by natural spawning, were maintained in E3 medium (5mM NaCl, 0.17mM KCl, 0.33mM CaCl_2_, 0.33mM MgSO_4_, 10^–5^ % methylene blue), and were staged according to hours post fertilization (hpf) and morphologic criteria [1]. Embryos euthanasia was performed by hypothermia shock for at least 20 minutes, while adults were euthanized by exposure to excess of tricaine methanesulfonate (MS-222, #A5040, Sigma).

**Plasmid cloning**

The plasmids injected in zebrafish embryos are the following:

pDEST(*mitfa:-2.3Hsa.BRAF_V600E-220,myl7:eGFP*) for the expression of BRAFV600E-ref cds;

pDEST(*mitfa:-2.3Hsa.BRAF_V600E-204,myl7:eGFP*) for the expression of BRAFV600E-X1 cds;

pDEST(*mitfa:-2.3Hsa.BRAF_V600E-X2,myl7:eGFP*) for the expression of BRAFV600E-X2 cds;

pDEST(*mitfa:-2.4Hsa.BRAF_V600E-220,myl7:eGFP*) for the expression of BRAFV600E-ref cds+3’UTR;

pDEST(*mitfa:-9.4Hsa.BRAF_V600E-204,myl7:eGFP*) for the expression of BRAFV600E-X1 cds+3’UTR.

They were generated using Tol2kit (http://tol2kit.genetics.utah.edu/index.php/Main_Page).

In brief, the cds sequence of human *BRAF* isoforms carrying the activating V600E mutation was amplified by PCR from PIG-BRAFV600E-ref, X1, and X2 plasmids [2]; the ref 3’UTR sequence was amplified from pMIR-ref-3’UTR [3]; the X1 3’UTR sequence was amplified from pCW-X1-3’UTR plasmid. In turn, pCW-X1-3’UTR plasmid was obtained by PCR amplification of the X1 3’UTR from A375 genomic DNA using 7kb_X1_3UTR_Fw (SalI STOP) primer (5’ agcgtcgacTAGCCACCATCATGGCAG 3’) and 7kb_X1_3UTR_Rv (MluI) primer (5’ CAGACGCGTttctccatgcagtcaatcttta 3’), and subsequent cloning in the pCW backbone [3] using SalI and MluI restriction enzymes. All human *BRAF* sequences are listed in **Supplementary material**. PCR amplicons were then inserted into the multiple cloning site of pME-MCS plasmid (Tol2kit), using SalI-SpeI and SpeI-NotI restriction sites. To create pDEST plasmids, pME-*BRAF* plasmids were mixed with p5E-*mitfa* promoter plasmid (kind gift from Dr. Charles Kaufman, Washington University School of Medicine, St. Louis, USA), p3E-polyA plasmid (Tol2kit), and pDestTol2CG* backbone plasmid (Tol2kit), in presence of Gateway™ LR Clonase™ Enzyme (Thermo Fisher Scientific), as reported in [4].

PCR reactions involved in cloning were performed using Phusion Flash High-Fidelity PCR Master Mix (Thermo Fisher Scientific) and the primers listed in **Suppl. Table 1**. PCR amplicons were run on a 0.8-2% agarose gel and extracted using QIAquick Gel Extraction Kit (Qiagen). Successful cloning was confirmed subjecting plasmids to Sanger sequencing (Eurofins Genomics).

**Plasmid microinjection in 1-cell stage zebrafish embryos**

Zebrafish of the *p53(lf)* strain (ZDB-ALT-050428-2) (kind gift from Dr. Francesco Argenton, Università di Padova) were bred and embryos were collected for microinjection. 25pg of plasmidic DNA and 25pg of *Tol2* mRNA were coinjected into 1-cell stage embryos for the biggest plasmid (pDEST(*mitfa:-9.4Hsa.BRAF_V600E-204,myl7:eGFP*)). Following the calculation of molar concentration, equimolar amounts of the other pDEST plasmids were microinjected. After microinjection, embryos were maintained in E3 medium at 28.5°C. At 24-48hpf they were selected based on heart-specific green fluorescence, using MZ10F Leica stereomicroscope. Successfully injected embryos were then subjected to further analysis, as described below.

**Zebrafish imaging and phenotypic analysis**

5dpf larvae were anesthetized and immersed in methylcellulose. Images were acquired using M80 Leica stereomicroscope, equipped with a Nikon DS-Fi1 camera.

Juvenile and adult fish were anesthetized, and images were acquired in water using M80 Leica stereomicroscope or ASUS Zenfone X00TD camera.

Nevi were defined as flat, strongly pigmented clusters of melanocytes that disrupt the distinctive striping pattern [5] [6]. Quantification of nevus area was performed using ImageJ software (<http://rsb.info.nih.gov>). For each animal, pictures of the lateral (right and left) and dorsal positions were analyzed. The area of the nevi visible in each picture was measured with ImageJ. Then, the biggest area for each animal was included in the graph and subjected to statistical analysis.

The transition from nevus to melanoma was detected as pigmentation intensification, which turns into skin thickening, accompanied by outward growth [7]. Melanoma tumors were defined as melanotic/amelanotic based on the presence/absence of pigmentation according to visual inspection (see **Suppl. Fig.7** for an example).

**Melanoma-free survival curves**

Adult animals up to 50 weeks old were checked weekly for the presence of melanoma tumors and Kaplan-Meier curves for melanoma-free survival were created.

**Collection, RNA extraction and retrotranscription of embryos and larvae**

Embryos and larvae (at least 20 per experimental condition) were homogenized by insulin needle. Total RNA was extracted using QIAzol (Qiagen) following the manufacturer’s instructions, quantified using Nanodrop Lite (Thermo Fisher Scientific), verified on 2% agarose gel and reverse transcribed with the SuperScript III RT reaction kit (Thermo Fisher Scientific) following the manufacturer’s instructions. The successful retrotranscription and the absence of contaminating genomic DNA were routinely checked through a control PCR (PCR Master Mix, Thermo Fisher Scientific) in which the exon-spanning primers for *actb1* mRNA are used.

**RT-PCR of embryos**

To detect the expression of *BRAFV600E* isoforms from pDEST plasmids (see above), GoTaq® G2 Green Master Mix (PROMEGA) was used with 10ng of cDNA, 0.5µM primers and 58°C annealing temperature. The primers list is reported in **Suppl. Table 1**.

**qRT-PCR of embryos and larvae**

Quantitative analysis of *BRAFV600E* isoforms expression from pDEST plasmids (see above) was performed in triplicate with SSOADV Universal SYBR Green (Bio-Rad) in 15µl final reaction volume on a CFX96 Real-Time System (Bio-Rad). 37.5ng of cDNA, 0.5uM primers and 60°C annealing temperature were used. The primers list is reported in **Suppl. Table 1**.

PCR efficiency and expression data were analyzed using CFX Manager Software (Bio-Rad). Relative expression of *BRAFV600E* isoforms was determined using the 2^−ΔΔCt^ method and data were normalized using housekeeping genes (*eef1a1l1, actb1*) [3].

**Collection and histological analysis of melanoma specimens**

Three weeks after tumor onset, fish were euthanized, then fixed in 4% PFA for 48h at 4°C, dehydrated through a series of graded ethanol baths, and finally embedded in paraffin. Transverse paraffin-embedded tissue sections (5µm) were used. Hematoxylin and Eosin (H&E) staining was carried out using standard methods. For immunohistochemistry (IHC) analysis, tissue sections were stained using standard whole-mount immunostaining protocol with Vectastain elite ABC kit (Vector Laboratories) and 1:50 mouse anti-BRAFV600E primary antibody (#ab228461, clone VE1, Abcam).

**Collection and western blot analysis of melanoma specimens**

Three weeks after tumor onset, fish were euthanized, and tumors were isolated. They were then homogenized with a pestle for 15-30 minutes, while kept on ice in 50-100uL of RIPA Buffer (50mM Tris HCl, 150mM NaCl, 0.5% NaDeoxicholate, 0.1% SDS, 1% NP40) supplemented with 1mM PMSF, 2mM Na orthovanadate, and cOmplete^™^ Protease Inhibitor (Roche). The mixture was centrifuged at 14000rpm for 30min at 4°C and the supernatant was quantified using BCA reagent (#23227, Thermo Fisher Scientific) at 590nm. 30µg of proteins were combined with 4X loading buffer (Bio-Rad), heated at 95°C for 5min, and loaded on a 4-15% SDS-polyacrylamide gel (Mini-PROTEAN Precast gel, BioRad) along with a molecular weight marker (Bio-Rad). Proteins were then electrotransferred to a polyvinylidene difluoride (PVDF) membrane using Trans-Blot Turbo system (Bio-Rad). Membranes were blocked at room temperature for 1h using 5% milk in TBST. They were then incubated overnight at 4°C with the following primary antibodies:

-mouse anti-BRAFV600E VE1 antibody ((#ab228461, clone VE1, Abcam; 1:400 dilution in 1% BSA in TBST);

-mouse anti-MCM7 monoclonal antibody (#sc-9966, clone 141.2, Santa Cruz Biotechnology; 1:200 dilution in 1% BSA in TBST);

-mouse anti-p-ERK 1/2 monoclonal antibody (#sc-7383, clone E-4, Santa Cruz Biotechnology; 1:500 dilution in 3% BSA in TBST);

-mouse anti-ERK 2 polyclonal antibody (#sc-1647, clone D-2, Santa Cruz Biotechnology; 1:500 dilution in 3% BSA in TBST).

Blots were washed 4 x 5min in TBST and incubated for 1h with the appropriate secondary antibody (1:3000 dilution in 5% milk in TBST). Blots were again washed 4 x 5min in TBST and developed using Clarity Western ECL blotting substrate (Bio-Rad). Finally, bands were detected using ChemiDoc imaging system (Bio-Rad). Membrane stripping was performed using Restore™ Western Blot Stripping Buffer (#21059, Thermo Fisher Scientific).

**Statistical analyses**

Data were analyzed according to their normality using parametric or non-parametric tests. qRT-PCR data were analyzed using one-way ANOVA (Tukey’s) test. Nevus percentage and tumor macro-features were analyzed using Fisher’s exact test. Nevus size was analyzed using Kruskal-Wallis (Dunn’s) test. Kaplan-Meier curves were analyzed using log-rank (Mantel-Cox) test. When appropriate, data are expressed as mean ± SEM (standard error of the mean). p < 0.05 was taken as a minimum level of significance. To account for biological and technical variability, at least 2 independent biological replicates were performed for each experiment. The total number of juvenile or adult fish studied for each experimental condition is reported in each graph.

**REFERENCES**

1. Kimmel CB, Ballard WW, Kimmel SR, Ullmann B, Schilling TF. Stages of embryonic development of the zebrafish. Developmental Dynamics. 1995;203:253–310.

2. Marranci A, Jiang Z, Vitiello M, Guzzolino E, Comelli L, Sarti S, et al. The landscape of BRAF transcript and protein variants in human cancer. Mol Cancer. 2017;16.

3. Marranci A, D’Aurizio R, Vencken S, Mero S, Guzzolino E, Rizzo M, et al. Systematic evaluation of the microRNAome through miR-CATCHv2.0 identifies positive and negative regulators of BRAF-X1 mRNA. RNA Biol. 2019;16.

4. Sarti S, De Paolo R, Ippolito C, Pucci A, Pitto L, Poliseno L. Inducible modulation of miR-204 levels in a zebrafish melanoma model. Biol Open. 2020;9.

5. Patton EE, Widlund HR, Kutok JL, Kopani KR, Amatruda JF, Murphey RD, et al. BRAF mutations are sufficient to promote nevi formation and cooperate with p53 in the genesis of melanoma. Curr Biol [Internet]. 2005/02/08. 2005;15:249–54. Available from: http://www.ncbi.nlm.nih.gov/pubmed/15694309

6. Dovey M, White RM, Zon LI. Oncogenic NRAS Cooperates with *p53* Loss to Generate Melanoma in Zebrafish. Zebrafish. 2009;6:397–404.

7. Patton EE, Mathers ME, Schartl M. Generating and analyzing fish models of melanoma. Methods Cell Biol [Internet]. 2011;105:339–66. Available from: https://www.ncbi.nlm.nih.gov/pubmed/21951537
