# Supplementary material for: Differential impact of BRAFV600E isoforms on tumorigenesis in a zebrafish model of melanoma
Source: Cell Biosci. 2023 Jul 1;13:121. doi: 10.1186/s13578-023-01064-w (PMC10314448; doi:10.1186/s13578-023-01064-w)
Supplement: Supplementary file 3 — Additional file 3. Supplementary material. Sequence of ref cds, X1 cds, X2 cds, ref 3’UTR, X1 3’UTR. [file 13578_2023_1064_MOESM3_ESM.docx]

**SUPPLEMENTARY MATERIAL. Sequence of ref cds, X1 cds, X2 cds, ref 3’UTR, X1 3’UTR.**

|  | Sequence 5’ 🡪 3’ |
| --- | --- |
| hBRAF-ref cds  (NM_004333.6) | ATGGCGGCGCTGAGCGGTGGCGGTGGTGGCGGCGCGGAGCCGGGCCAGGCTCTGTTCAACGGGGACATGGAGCCCGAGGCCGGCGCCGGCGCCGGCGCCGCGGCCTCTTCGGCTGCGGACCCTGCCATTCCGGAGGAGGTGTGGAATATCAAACAAATGATTAAGTTGACACAGGAACATATAGAGGCCCTATTGGACAAATTTGGTGGGGAGCATAATCCACCATCAATATATCTGGAGGCCTATGAAGAATACACCAGCAAGCTAGATGCACTCCAACAAAGAGAACAACAGTTATTGGAATCTCTGGGGAACGGAACTGATTTTTCTGTTTCTAGCTCTGCATCAATGGATACCGTTACATCTTCTTCCTCTTCTAGCCTTTCAGTGCTACCTTCATCTCTTTCAGTTTTTCAAAATCCCACAGATGTGGCACGGAGCAACCCCAAGTCACCACAAAAACCTATCGTTAGAGTCTTCCTGCCCAACAAACAGAGGACAGTGGTACCTGCAAGGTGTGGAGTTACAGTCCGAGACAGTCTAAAGAAAGCACTGATGATGAGAGGTCTAATCCCAGAGTGCTGTGCTGTTTACAGAATTCAGGATGGAGAGAAGAAACCAATTGGTTGGGACACTGATATTTCCTGGCTTACTGGAGAAGAATTGCATGTGGAAGTGTTGGAGAATGTTCCACTTACAACACACAACTTTGTACGAAAAACGTTTTTCACCTTAGCATTTTGTGACTTTTGTCGAAAGCTGCTTTTCCAGGGTTTCCGCTGTCAAACATGTGGTTATAAATTTCACCAGCGTTGTAGTACAGAAGTTCCACTGATGTGTGTTAATTATGACCAACTTGATTTGCTGTTTGTCTCCAAGTTCTTTGAACACCACCCAATACCACAGGAAGAGGCGTCCTTAGCAGAGACTGCCCTAACATCTGGATCATCCCCTTCCGCACCCGCCTCGGACTCTATTGGGCCCCAAATTCTCACCAGTCCGTCTCCTTCAAAATCCATTCCAATTCCACAGCCCTTCCGACCAGCAGATGAAGATCATCGAAATCAATTTGGGCAACGAGACCGATCCTCATCAGCTCCCAATGTGCATATAAACACAATAGAACCTGTCAATATTGATGACTTGATTAGAGACCAAGGATTTCGTGGTGATGGAGGATCAACCACAGGTTTGTCTGCTACCCCCCCTGCCTCATTACCTGGCTCACTAACTAACGTGAAAGCCTTACAGAAATCTCCAGGACCTCAGCGAGAAAGGAAGTCATCTTCATCCTCAGAAGACAGGAATCGAATGAAAACACTTGGTAGACGGGACTCGAGTGATGATTGGGAGATTCCTGATGGGCAGATTACAGTGGGACAAAGAATTGGATCTGGATCATTTGGAACAGTCTACAAGGGAAAGTGGCATGGTGATGTGGCAGTGAAAATGTTGAATGTGACAGCACCTACACCTCAGCAGTTACAAGCCTTCAAAAATGAAGTAGGAGTACTCAGGAAAACACGACATGTGAATATCCTACTCTTCATGGGCTATTCCACAAAGCCACAACTGGCTATTGTTACCCAGTGGTGTGAGGGCTCCAGCTTGTATCACCATCTCCATATCATTGAGACCAAATTTGAGATGATCAAACTTATAGATATTGCACGACAGACTGCACAGGGCATGGATTACTTACACGCCAAGTCAATCATCCACAGAGACCTCAAGAGTAATAATATATTTCTTCATGAAGACCTCACAGTAAAAATAGGTGATTTTGGTCTAGCTACAGTGAAATCTCGATGGAGTGGGTCCCATCAGTTTGAACAGTTGTCTGGATCCATTTTGTGGATGGCACCAGAAGTCATCAGAATGCAAGATAAAAATCCATACAGCTTTCAGTCAGATGTATATGCATTTGGGATTGTTCTGTATGAATTGATGACTGGACAGTTACCTTATTCAAACATCAACAACAGGGACCAGATAATTTTTATGGTGGGACGAGGATACCTGTCTCCAGATCTCAGTAAGGTACGGAGTAACTGTCCAAAAGCCATGAAGAGATTAATGGCAGAGTGCCTCAAAAAGAAAAGAGATGAGAGACCACTCTTTCCCCAAATTCTCGCCTCTATTGAGCTGCTGGCCCGCTCATTGCCAAAAATTCACCGCAGTGCATCAGAACCCTCCTTGAATCGGGCTGGTTTCCAAACAGAGGATTTTAGTCTATATGCTTGTGCTTCTCCAAAAACACCCATCCAGGCAGGGGGATATGGTGCGTTTCCTGTCCACTGA |
| hBRAF-X1 cds  (NM_001354609.2) | ATGGCGGCGCTGAGCGGTGGCGGTGGTGGCGGCGCGGAGCCGGGCCAGGCTCTGTTCAACGGGGACATGGAGCCCGAGGCCGGCGCCGGCGCCGGCGCCGCGGCCTCTTCGGCTGCGGACCCTGCCATTCCGGAGGAGGTGTGGAATATCAAACAAATGATTAAGTTGACACAGGAACATATAGAGGCCCTATTGGACAAATTTGGTGGGGAGCATAATCCACCATCAATATATCTGGAGGCCTATGAAGAATACACCAGCAAGCTAGATGCACTCCAACAAAGAGAACAACAGTTATTGGAATCTCTGGGGAACGGAACTGATTTTTCTGTTTCTAGCTCTGCATCAATGGATACCGTTACATCTTCTTCCTCTTCTAGCCTTTCAGTGCTACCTTCATCTCTTTCAGTTTTTCAAAATCCCACAGATGTGGCACGGAGCAACCCCAAGTCACCACAAAAACCTATCGTTAGAGTCTTCCTGCCCAACAAACAGAGGACAGTGGTACCTGCAAGGTGTGGAGTTACAGTCCGAGACAGTCTAAAGAAAGCACTGATGATGAGAGGTCTAATCCCAGAGTGCTGTGCTGTTTACAGAATTCAGGATGGAGAGAAGAAACCAATTGGTTGGGACACTGATATTTCCTGGCTTACTGGAGAAGAATTGCATGTGGAAGTGTTGGAGAATGTTCCACTTACAACACACAACTTTGTACGAAAAACGTTTTTCACCTTAGCATTTTGTGACTTTTGTCGAAAGCTGCTTTTCCAGGGTTTCCGCTGTCAAACATGTGGTTATAAATTTCACCAGCGTTGTAGTACAGAAGTTCCACTGATGTGTGTTAATTATGACCAACTTGATTTGCTGTTTGTCTCCAAGTTCTTTGAACACCACCCAATACCACAGGAAGAGGCGTCCTTAGCAGAGACTGCCCTAACATCTGGATCATCCCCTTCCGCACCCGCCTCGGACTCTATTGGGCCCCAAATTCTCACCAGTCCGTCTCCTTCAAAATCCATTCCAATTCCACAGCCCTTCCGACCAGCAGATGAAGATCATCGAAATCAATTTGGGCAACGAGACCGATCCTCATCAGCTCCCAATGTGCATATAAACACAATAGAACCTGTCAATATTGATGACTTGATTAGAGACCAAGGATTTCGTGGTGATGGAGGATCAACCACAGGTTTGTCTGCTACCCCCCCTGCCTCATTACCTGGCTCACTAACTAACGTGAAAGCCTTACAGAAATCTCCAGGACCTCAGCGAGAAAGGAAGTCATCTTCATCCTCAGAAGACAGGAATCGAATGAAAACACTTGGTAGACGGGACTCGAGTGATGATTGGGAGATTCCTGATGGGCAGATTACAGTGGGACAAAGAATTGGATCTGGATCATTTGGAACAGTCTACAAGGGAAAGTGGCATGGTGATGTGGCAGTGAAAATGTTGAATGTGACAGCACCTACACCTCAGCAGTTACAAGCCTTCAAAAATGAAGTAGGAGTACTCAGGAAAACACGACATGTGAATATCCTACTCTTCATGGGCTATTCCACAAAGCCACAACTGGCTATTGTTACCCAGTGGTGTGAGGGCTCCAGCTTGTATCACCATCTCCATATCATTGAGACCAAATTTGAGATGATCAAACTTATAGATATTGCACGACAGACTGCACAGGGCATGGATTACTTACACGCCAAGTCAATCATCCACAGAGACCTCAAGAGTAATAATATATTTCTTCATGAAGACCTCACAGTAAAAATAGGTGATTTTGGTCTAGCTACAGAGAAATCTCGATGGAGTGGGTCCCATCAGTTTGAACAGTTGTCTGGATCCATTTTGTGGATGGCACCAGAAGTCATCAGAATGCAAGATAAAAATCCATACAGCTTTCAGTCAGATGTATATGCATTTGGGATTGTTCTGTATGAATTGATGACTGGACAGTTACCTTATTCAAACATCAACAACAGGGACCAGATAATTTTTATGGTGGGACGAGGATACCTGTCTCCAGATCTCAGTAAGGTACGGAGTAACTGTCCAAAAGCCATGAAGAGATTAATGGCAGAGTGCCTCAAAAAGAAAAGAGATGAGAGACCACTCTTTCCCCAAATTCTCGCCTCTATTGAGCTGCTGGCCCGCTCATTGCCAAAAATTCACCGCAGTGCATCAGAACCCTCCTTGAATCGGGCTGGTTTCCAAACAGAGGATTTTAGTCTATATGCTTGTGCTTCTCCAAAAACACCCATCCAGGCAGGGGGATATGGAGAATTTGCAGCCTTCAAGTAG |
| hBRAF-X2 cds  (NM_001378468.1) | ATGGCGGCGCTGAGCGGTGGCGGTGGTGGCGGCGCGGAGCCGGGCCAGGCTCTGTTCAACGGGGACATGGAGCCCGAGGCCGGCGCCGGCGCCGGCGCCGCGGCCTCTTCGGCTGCGGACCCTGCCATTCCGGAGGAGGTGTGGAATATCAAACAAATGATTAAGTTGACACAGGAACATATAGAGGCCCTATTGGACAAATTTGGTGGGGAGCATAATCCACCATCAATATATCTGGAGGCCTATGAAGAATACACCAGCAAGCTAGATGCACTCCAACAAAGAGAACAACAGTTATTGGAATCTCTGGGGAACGGAACTGATTTTTCTGTTTCTAGCTCTGCATCAATGGATACCGTTACATCTTCTTCCTCTTCTAGCCTTTCAGTGCTACCTTCATCTCTTTCAGTTTTTCAAAATCCCACAGATGTGGCACGGAGCAACCCCAAGTCACCACAAAAACCTATCGTTAGAGTCTTCCTGCCCAACAAACAGAGGACAGTGGTACCTGCAAGGTGTGGAGTTACAGTCCGAGACAGTCTAAAGAAAGCACTGATGATGAGAGGTCTAATCCCAGAGTGCTGTGCTGTTTACAGAATTCAGGATGGAGAGAAGAAACCAATTGGTTGGGACACTGATATTTCCTGGCTTACTGGAGAAGAATTGCATGTGGAAGTGTTGGAGAATGTTCCACTTACAACACACAACTTTGTACGAAAAACGTTTTTCACCTTAGCATTTTGTGACTTTTGTCGAAAGCTGCTTTTCCAGGGTTTCCGCTGTCAAACATGTGGTTATAAATTTCACCAGCGTTGTAGTACAGAAGTTCCACTGATGTGTGTTAATTATGACCAACTTGATTTGCTGTTTGTCTCCAAGTTCTTTGAACACCACCCAATACCACAGGAAGAGGCGTCCTTAGCAGAGACTGCCCTAACATCTGGATCATCCCCTTCCGCACCCGCCTCGGACTCTATTGGGCCCCAAATTCTCACCAGTCCGTCTCCTTCAAAATCCATTCCAATTCCACAGCCCTTCCGACCAGCAGATGAAGATCATCGAAATCAATTTGGGCAACGAGACCGATCCTCATCAGCTCCCAATGTGCATATAAACACAATAGAACCTGTCAATATTGATGACTTGATTAGAGACCAAGGATTTCGTGGTGATGGAGGATCAACCACAGGTTTGTCTGCTACCCCCCCTGCCTCATTACCTGGCTCACTAACTAACGTGAAAGCCTTACAGAAATCTCCAGGACCTCAGCGAGAAAGGAAGTCATCTTCATCCTCAGAAGACAGGAATCGAATGAAAACACTTGGTAGACGGGACTCGAGTGATGATTGGGAGATTCCTGATGGGCAGATTACAGTGGGACAAAGAATTGGATCTGGATCATTTGGAACAGTCTACAAGGGAAAGTGGCATGGTGATGTGGCAGTGAAAATGTTGAATGTGACAGCACCTACACCTCAGCAGTTACAAGCCTTCAAAAATGAAGTAGGAGTACTCAGGAAAACACGACATGTGAATATCCTACTCTTCATGGGCTATTCCACAAAGCCACAACTGGCTATTGTTACCCAGTGGTGTGAGGGCTCCAGCTTGTATCACCATCTCCATATCATTGAGACCAAATTTGAGATGATCAAACTTATAGATATTGCACGACAGACTGCACAGGGCATGGATTACTTACACGCCAAGTCAATCATCCACAGAGACCTCAAGAGTAATAATATATTTCTTCATGAAGACCTCACAGTAAAAATAGGTGATTTTGGTCTAGCTACAGTGAAATCTCGATGGAGTGGGTCCCATCAGTTTGAACAGTTGTCTGGATCCATTTTGTGGATGGCACCAGAAGTCATCAGAATGCAAGATAAAAATCCATACAGCTTTCAGTCAGATGTATATGCATTTGGAATTGTTCTGTATGAATTGATGACTGGACAGTTACCTTATTCAAACATCAACAACAGGGACCAGATAATTTTTATGGTGGGACGAGGATACCTGTCTCCAGATCTCAGTAAGGTACGGAGTAACTGTCCAAAAGCCATGAAGAGATTAATGGCAGAGTGCCTCAAAAAGAAAAGAGATGAGAGACCACTCTTTCCCCAAGAGAATTTGCAGCCTTCAAGTAGCCACCATCATGGCAGCATCTGCTCTTATTTCTTAAGTCTTGTGTTCGTACAATTTGTTAACATCAAAACACAGTTCTGTTCCTCAAATCTTTTTTTAAAGATACAAAATTTCCAATGCATAAGCTGA |
| hBRAF-ref 3’UTR  (NM_004333.6) | aacaaatgagtgagagagttcaggagagtagcaacaaaaggaaaataaatgaacatatgtttgcttatatgttaaattgaataaaatactctctttttttttaaggtgaaccaaagaa |
| hBRAF-X1 3’UTR  (NM_001354609.2) | ccaccatcatggcagcatctgctcttatttcttaagtcttgtgttcgtacaatttgttaacatcaaaacacagttctgttcctcaaatctttttttaaagatacaaaatttccaatgcataagctgatgtggaacagaatggaatttcccatccaacaaaagaggaaagaatgttttaggaaccagaattctctgctgccagtgtttcttcaacaaaaataccacgagcatacaagtctgcccagtcccaggaagaaagaggagagaccctgaattctgaccttttgatggtcaggcatgatggaaagaaactgctgctacagcttgggagatttgctatggaaagtctgccagtcaactttgcccttctaaccaccagatcaatttgtggctgatcatctgatggggcagtttcaatcaccaagcatcgttctctttcctgttctggaattttgttttggagctctttcccctagtgaccaccagttagtttctgagggatggaacaaaaatgcagcttgccctttctatgtggtgcgtgttcaggccttgacagattttatcaaaaggaaactattttatttaaatggaggctgagtggtgagtagatgtgtcttggtatggaggaaaagggcatgctgcatcttcttcctgacctccggggtctctggccttttgtttccttgctcactgaggggtctgtctaaccaagcaggctagatagtgctggcacacattgccttctttctcattgggtccagcaatgaagataagtgtttgggttttttttttttcctccacaatgtagcaaattctcaggaaatacagtttatatcttcctcctatgctcttccagtcaccaactacttatgcggctactttgtccagggcacaaaatgccgtggcagtatctaactaaacccccacaaaactgcttaataacagttttgaatgtgagaaatttagataatttaaatataaggtacaggttttaatttctgagtttcttcttttctatttttattaaaaagaaaataattttcagatttaattgaattggaaaaaaacaatacttcccaccagaattatatatcctgaaaattgtatttttgttatataaacaacttttaagaaagatcattatccttttctctacctaaatatgaggagtcttagcataatgacaaatatttataatttttcaattaatggtacttgctggatccacactaacatctttgctaataatctcattgtttcttccaactgattcctaacactatatcccacatcttctttctagtcttttatctagaatatgcaacctaaaataaaaatggtggcgtctccattcattctccttcttccttttttcccaagcctggtcttcaaaaggttgggcaatttggcagctgaattcccagacagagaatagagcaattttagggatattaggactgagggagggtgtgggaaagctgtcatcagttgtttttatagaaagaactggcattcattaagaacctaaatcttatctttgcacaaatggaaaatataacctagttatagcttcctttggcctttattaaagggtaatatcaatcacagtcatagcaaagaaagcggatgtattaatggcaaattaatggaaaacctcccttatcaggaatctagactcagaatttaggaacacaaatcaaatcagaccaaccaagctatagccaaggacttgaaagaaattaaacaagacccagaataaatcaaggaattagaaattgttatttaaaaatttcagattgtaactccaggccctgctgtctatattgcagccactaaaagctcactaccattagatttttgctaacatacatgtattcagaagaaagcctattgaaattttcattgtcttgtaaaaggttgtcctagtaaaatggaaaagatccttaagttattaatcagtttgaaaagcaaatttgtttttaagttttacatcagcagggcagtgtcttacaaaattcagaaattgcaaaggtggaaataattcacgctgatttgaagaacatcttctgtgcaataatactgcctctcttgaaaagcattggctgttttttctttttaaatatatctctagatgcttttaaatgtggctgtgttccctttaccaagattggcttcaagtttccgcaggtagagagacctgggcttgaacaagaggatgtgtttcatgtcctgctgaggaggtagaacatgtgcagcctgggtccgggactgcctccgtggggcaggggcaggggcggtaccattagggaggaagcttagcatttcagtttcttaaacaatattcagggtgatacactttttcttcccttgcattttagaataggctggtatctcatttgaacgggggagcagacttgatctcaaatgaagctgtgcccaggagccaggcttagcatattgagatttttatagataccttaaaaaataaaatatttaaacctctcttttcttcctttttctatgaaataggttttttctctagtttacaaatgacatgaaaataggttttatttgtgttttatctgctttattttttgatgcttagacaacagttagacttactgagctcctaaaaaaacgaggaagaagtccttatttgtgaaaagcactttatgagtaattgtatagacagtatgtggctgcgtcactgatcatcttgtaagggtgtaacagtcttgtctgtaaagtggctgcagtgccttctgtagtgtgttttatttttggtagggagaggtgaagccttctgaaaaatttgagagcaactacagaggattgtttgtaactgtgtagtattcctgatggacttttttcatcgttagagtcaaggacctagacttttgccactgaaataatattgaccaaaaaaatagtttataaaagggatttgtgaatagaaaattcagtgtgatcatttgttgttaatgtgcaccttaaaagaagattctgtctagctgtcaaattctggttcccgaatatctcacccctgattgtatttgagatctagtagggcatactggggcattttagaagataaaatcccatacaaatgatatatgctatatttatgttggtgttggagaagaaagagcagtatataaagaaataattcaagactgcagcactgtcaacctgaaactttgtaaatatttcctagcttctggtttggtgcggtgacagcactttcatcacaggatgttaccttgtattcaccaggcggagtgcgagctgctgcacatcctcctcagatctcacctgtccccactgtacatccacccgccagctgcttgcaaacctcatctctagctttagttcgaaaccacattgcagggttcaggtgacctctacaaaaaactacctcttcagaatgaggtaatgaatagttatttattttaaaatatgaaaagtcaggagctctagaacatgacgatgatttaagattttaacttttttgtgtacttgtatttgagcactctcattttgtcctaaagggcattatacatttaagcagtaatactgtaaaaaaatgtgttgctcggaatatctgaatgttgttgaaagtggtgccagaaccggtttaggggtacgtttcagaatcttaaccttgagtcaattgcatgaaattaaatagctgtggtatcacttcactaacagtgatgtaattttaattttcagtaggcttggcatgacagtacatcctcataatgagtttgctgcagctttgtcacatgcacaggcattcatagaaagaccacccagctaagagggtagaatgattactctttttgcaagattctcttctttgtccaagttggcattgttagtgctaggaataccagcaccttgagacgagcagattccaaccattaggctataaacaccatagccagagatggaaggtttactgtgagtatgaacagcaaatagcttacaggtcatgagttgaaatggtgtaggtgaggctctagaaaaataccttgacaatttgccaaatgatcttactgtgccttcatgatgcaataaaaaagctaacattttagcagaaatcagtgatttgtgaagagagcagccactctggtttaactcagctgtgttaataatttttagagtgcaatttagactgcataggtaaatgcactaaagagtttatagccaaaatcacatttaacaatgagaaaacacacaggtaaattttcagtgaacaaaattatttttttaaagcacataatccctagtatagtcagatatatttatcacatagagcaactaggttgcaaatatagttcagtgacatttctagagaaactttttctactcccataggctcttcaaagcatggaacttttatacaacagaaatgttgacagaaattgctgtagtttagggttgaagtactgtatgatgggcagcaatcatgtattaacttagaaggggaaattgaaatataggaccgaatttggttttatcagtttccagagtactgctgccaacctagacactgatttttcagagtttgaaatgtaaatttcttcccgggacttgattgcacatgaagctggactgcgttagtcatcctgtcccaaagcgctgtgggggccagggtggaggtctcaaggcatcctttatgacctggccattggatgtaaaagaaaacatattccatgctgtggttcttgtatcttgtttcattcctcaccattgaaagagaaagtccatgtattgtctccagcacatccttgaaatgttatactgggatggattactgatgcccatcggtagttgagccccagaagagggtagtagcatctctgcctcaggtgatgatttgtagcttggccagaggagagcggagtcaccagtatatctgtggtccatgttgctagctctggtaaaattaaaaatactggtaagatgtttgttttattagtacactagacagtaagctctgttttgttgttttcaaataacctattttcacttttgtttgggcaaagacatttaaattgaaattcaattctaatttttgttaattgtggaaagggtaattaacagttcctatcaggtatttttaatgtggaaaaggacagaaacccaactcctaaaatcttaaattaaggtaacagtgctttaaaaaaaaaaaatgcatggggcaattagtcggcaactcaatgagtgactaaagtacttttatttaacatccacaacttcaactgttaagttttattaattactaaatcagctttattaaaatgttgacatttatttagctattttgaataattatagtgacttgacgagtgtgtatgaggacacagccaatgtaagccagtgtatccattttttagaggtgcatttttttttaaagaattctgtagatagaagtgctctgaaaacaactaaaatatgtttattcatggtagtatcaaaaaatgtttgtacaaaccatctgcttctcccggccagccgagttcattctccagcaccgtgaccgctggttctcatgtacagcacatatgcgggagagttggcagaaaatttgtgaagagatgccgcaaaggaagggtctgttgacgggtgggattgggggttttgatgaagttgcttagtcctggttttgttttgaaaattactgcgttgcatttttgtgttaagtttttgaacccacgtgtgttttggtggagtatgagttggaagtcactgcaaactagcataaacaacaaagctcacagagtaggcacagatgtagagaacagagaccaaaatggggtgaggtggcagtaaatctaggatagggaaaaattaatgtgagggtgggaaataaactgtaattacctgaaatcaaatgtaagagtgcaataagtatgctttttattctaagctgtgaacggtttttttaagaatcattccttcctaatacatttgtgtatgttccatagctgattaaaaccagctatatcaacatataatgcctttttattcatgttaatgaccaacgtaagtggctagcctttatgtcttatttatcttcatgttatgttagtttacatacaggggtgtatgtctctgtgctgtccccttctcctgccttcattttaaaatgcatccatgggtcctccgtgtttcctttggccatgccacatatatagactcagtttggccttcatgatatcgcctgatttttgaggactgtatcacagtgatatgtatttgtggtaatctcatttgttggttgtacatctgatcctttcctcaacatggcaattgctgcctttcctaagataggatcatacaactgatcaggggattgaatttgatcattcatcaacatgtgtctctgaattttattcagtagttgtcattgctctttggtttagaccaagaaaaaggaaatccccccttttcatgtattccttggtttgaggacatgactcctgtaagggagaggaaagggagatgcttcctgtttgaactgcagtgaattcacggttcctgtttcaccactccaaaccttatggcgactcacacacacattcctcttttctgttactgccaaaggttcgggtttagtacacttcagttccactcaagcattgaaaaggttctcgtggagtctggggcgtgcccagtgaaaagatggggactttttaattgtccacagacctctctatacctgctttgcaaaaattacaatggagtaactatttttaaagcttatttttcaattcataaaaaagacatttattttcagtcaaatggatgatgtctccctcttttcccctattctcaatgtttgcttgaatcttttattattttttttaattctcccccatacccacttcctgatactttggttctctttcctgctcaggtcccttcatttgtactttggagtttttctcatgtaaatttgtataacagaaaatattgttcagtttggatagaaagcatggagaataaaaaaagatagctgaaattcagattgaagaaatttatttctgtgtaaagttatttaaaaactgtattatataaaaggcaaaaaaagttctatgtacttgatgtgaatatgcgaatactgctataataaagattgactgcatggagaa |
