# Supplementary material for: Differential impact of BRAFV600E isoforms on tumorigenesis in a zebrafish model of melanoma
Source: Cell Biosci. 2023 Jul 1;13:121. doi: 10.1186/s13578-023-01064-w (PMC10314448; doi:10.1186/s13578-023-01064-w)
Supplement: Supplementary file 4 — Additional file 4. Supplementary Table 1. Primer sequence and use. [file 13578_2023_1064_MOESM4_ESM.docx]

**Supplementary Table 1. Primer sequence and use.**

| Primer | Sequence 5’ 🡪 3’ | Use |
| --- | --- | --- |
| hBRAF-cds common-SalI -Kozak sequence -Fw | ATAGTCGACGCCACCATGGCGGCGCTGAGCGGT | hBRAF cds cloning |
| hBRAF-ref cds SpeI stop codon Rev | ATAACTAGTTCAGTGGACAGGAAACG | hBRAF-ref cds cloning |
| hBRAF-ref X1SpeI stop codon Rev | ATAACTAGTCTACTTGAAGGCTGCAAATTCTC | hBRAF-X1 cds cloning |
| hBRAF-ref X2 SpeI stop codon Rev | ATAACTAGTTCAGCTTATGCATTGGAAATT | hBRAF-X2 cds cloning |
| hBRAF-ref 3’UTR SpeI Fw | ATAACTAGTTGAAACAAATGAGTGAGAGAG | hBRAF-ref 3’UTR cloning |
| hBRAF-ref 3’UTR NotI Rev | ATAGCGGCCGCTTCTTTGGTTCACCTTAA |  |
| hBRAF-X1 3’UTR SpeI Fw | ATAACTAGTTAGCCACCATCATGGCAGCATC | hBRAF-X1 3’UTR cloning |
| hBRAF-X1 3’UTR NotI Rev | ATAGCGGCCGCTTCTCCATGCAGTCAATCT |  |
| hBRAFcds_377 Fw | CTAGCCTTTCAGTGCTACCTTCATCT | common cds  RT-PCR |
| hBRAFcds_1001_Rev | GGACTGGTGAGAATTTGGGGC |  |
| Ex14_1705 Fw | GCCAAGTCAATCATCCACAG | ref cds specific RT-PCR |
| hBRAFcdsRef_2300_Rev | CAGTGGACAGGAAACGCACCATAT |  |
| Ex15_1841 Fw | CTGGATCCATTTTGTGGATG | X1/X2 cds specific (ex19) RT-PCR |
| hBRAFcdsX1_2300_Rev | CTTGAAGGCTGCAAATTCT |  |
| Ex17_2075 Fw | TAATGGCAGAGTGCCTCAAA | ref cds-3’UTR junction specific RT-PCR |
| hBRAFutrRef_38 Rev | TGTTGCTACTCTCCTGAACTC |  |
| X1 only qRT_2179 Fw | AGTGCATCAGAACCCTCCTT | X1 cds-3’UTR junction specific RT-PCR |
| hBRAFutrX1_387 Rev | TTGATCTGGTGGTTAGAAGGG |  |
| hBRAFutrX1_6041Fw | TGTTAATGACCAACGTAAGTGGC | X1 3’UTR end specific RT-PCR |
| hBRAFutrX1_7155 Rev | GCAGTCAATCTTTATTATAGCAG |  |
| actb1_Intr311_Fw | TCAGGGAGTGATGGTTGGC | RNA control, exon spanning RT-PCR |
| actb1_ Rev | CAACGGAAACGCTCATTGC |  |
| eef1a1l1_Fw | GTACTTCTCAGGCTGACTGTG | Housekeeping qRT-PCR |
| eef1a1l1_Rev | ACGATCAGCTGTTTCACTCC |  |
| actb1_ Fw | TGAGCAGGAGATGGGAACC | Housekeeping qRT-PCR |
| actb1_ Rev | CAACGGAAACGCTCATTGC |  |
| hBRAFcds_377 Fw | CTAGCCTTTCAGTGCTACCTTCATCT | Hsa specific/common coding BRAF qRT-PCR |
| hBRAF-qRT1 Rev | TCCGTGCCACATCTGTGGGAT |  |
